# The co-occurrence of the SAVA syndemic, depression and anxiety as barriers to antiretroviral therapy adherence among sub-Saharan Africa population groups: A scoping review protocol

**DOI:** 10.1371/journal.pone.0274614

**Published:** 2022-09-20

**Authors:** Anton Delport, Hanani Tabana, Lucia Knight, Edwin Wouters

**Affiliations:** 1 School of Public Health, University of the Western Cape, Bellville, South Africa; 2 Division of Social and Behavioural Sciences, University of Cape Town, Cape Town, South Africa; 3 Department of Sociology, University of Antwerp, Antwerpen, Belgium; PLOS (Public Library of Science), UNITED KINGDOM

## Abstract

**Introduction:**

The scale-up of access to antiretroviral therapy has transformed HIV from an acute, terminal disease to a manageable chronic illness. Yet, sustaining high levels of antiretroviral therapy adherence remain a challenge, especially in the sub-Saharan Africa region which is disproportionately affected by HIV. This protocol proposes a scoping review to explore literature reporting on the antiretroviral therapy adherence levels among people who experience substance abuse and violence (SAVA) syndemics, as well as mood disorders such as anxiety and depression among people living with HIV in sub-Saharan Africa.

**Methods and analysis:**

This proposed scoping review will follow Arksey and O’Malley’s methodological framework for conducting scoping reviews as refined by Levac *et al*. The review will follow the Joanna Briggs Institute’s manual for conducting scoping reviews. Literature searches will be conducted using six databases: Academic search complete; APA PsycArticles; CINAHL; MEDLINE; SocINDEX and Web of science. Title screening will see the “Participant, Concept, Context” framework applied to identify relevant literature and will not include the appraisal of search results. Data charting will follow an adapted version of Trico and colleagues’ PRISMA-ScR and results will be mapped descriptively and in tabular format. Furthermore, results will be discussed within the syndemics model of health, and summarised as a biosocial conceptual model.

**Ethics and dissemination:**

The study will make use of secondary data that are readily available to the public and will not require ethical approval. We intend to publish our results in a peer-reviewed journal and disseminate our findings at relevant conferences and seminars.

## Introduction

The human immunodeficiency virus (HIV) remains a major global health epidemic with an estimated 38 million people living with HIV (PLWH), 1.7 million of whom were newly infected in 2020 [1]. Sub-Saharan Africa (SSA) is disproportionately affected by HIV as the region currently holds almost two thirds of the global HIV burden [2] and more than 60% of new infections [3]. The scale-up of access to antiretroviral therapy (ART) have transformed HIV from an acute, terminal disease to a manageable chronic illness [4, 5]. Rigid ART adherence behaviour has been shown to significantly decrease viral loads in PLWH to undetectable levels, effectively eliminating the risk of transmission [6]. Furthermore, good adherence to ART, meaning, taking antiretroviral medication as prescribed by a health practitioner, prevents immunological damage and improves life expectancy [7, 8].

It is for these reasons that it is essential to reach and sustain high levels of good ART adherence, however, it remains challenging for SSA–one of the poorest regions in the world [9]. There are social challenges which potentially not only induce the spread of HIV, but also hamper ART adherence. For example, the robust link between poverty and violence [10] and sexual and gender-based violence (SGBV) places women and young girls in particular at risk [11]. Studies have found that indirect exposure to violence [12] or being the recipient of violence [13] could significantly hinder an individual’s capacity to adhere to ART [14].

Violence is often accompanied by substance use [15] with a particular high prevalence among women [16]. The literature highlights the intersecting, co-occurring and mutually reinforcing factors of substance use and violence as determinants of HIV risk [17] and non-adherence behaviour [18]. This distinguished phenomenon is known as the SAVA syndemic, a mnemonic that stands for Substance Abuse, Violence and HIV/AIDS [19]. This syndemic model closely considers the details of biosocial factors and how these through their interaction perpetuate adverse health outcomes [19] and impede sustainable ART success.

When studying the link between the SAVA syndemic and ART adherence, an important correlate linked to ART adherence factors come into play; namely mood disorders. Mood disorders, in terms of multimorbidity of disease, often occur as an outcome of-, or as a pretext to violence [19] and/or substance use [20]. This relationship of adverse health outcomes has been well documented in the literature; with depression and anxiety being the most common mood disorders associated with violence [21] and substance use [22]. Furthermore, depression and anxiety are typical mood disorders found among PLWH, and are considered to be key barriers to ART adherence and hamper positive, or ‘good’ adherence behaviour [23]. Thus, it can be argued that when assessing the impact of the SAVA syndemic on ART adherence levels, the influence that stems from co-occurring mood disorders, and in particular depression and anxiety, should also be accounted for.

### Study rationale

Health problems rarely exist independently, and exploring the interplay between health problems, how they cluster and mutually impact ART adherence behaviour, is crucial in understanding and managing the spread of HIV. It is important to recognize that the SAVA syndemic is further confounded by mood disorders which typically manifest as anxiety and depression. The presence of both the SAVA syndemic and mood disorders have been found to be independent- and co-occurring as mutually re-enforcing barriers to ART adherence. Contemporary literature fails to adequately address the existence of mood disorders as co-occurring health burdens, and should be included when assessing the impact of the SAVA syndemic on ART adherence behaviour. This scoping review will attempt to bridge this gap by exploring the most recent literature around this topic and offer new insights into the biosocial factors that reinforce poor ART adherence levels.

### Theoretical underpinning of the study

The intended study aims to investigate secondary data around adverse biosocial factors common among PLWH, and how these factors impact individual capacity to manage HIV disease progression. Because of the aforementioned, we will discuss our results through the syndemics model of health [19]. This theoretical lens regards adverse biosocial factors as interacting, mutually occurring and reinforcing diseases; which flourishes under negative social and environmental factors [19]. The theory considers individual epidemics as sustained within communities due to harmful social connections, interactions and/or conditions [24]. It accounts for multiple interrelated systems that may contribute [24] to PLWHs poor levels of adherence behaviour by unpacking the syndemic interactions of substance use, IPV and poor mental health.

### Aims and objectives

The study aims to explore literature reporting on substance use, violence, anxiety and depression among PLWH in SSA. We intend to identify the characteristics of these factors and map the outcomes as thematic domains of mutually occurring health problems which may synergistically impede ART adherence behaviour among SSA populations. The following objectives will guide the study:

1. Map the prevalence of the SAVA syndemic among PLWH on ART in SSA
2. Map the coexistence of the SAVA syndemic and depression/anxiety among PLWH on ART in SSA
3. Explore impact of the SAVA syndemic on ART adherence in SSA
4. Explore the impact of the combination of SAVA and depression/anxiety on ART adherence in SSA

## Methods

### Scoping review methodology

**Fig 1:**
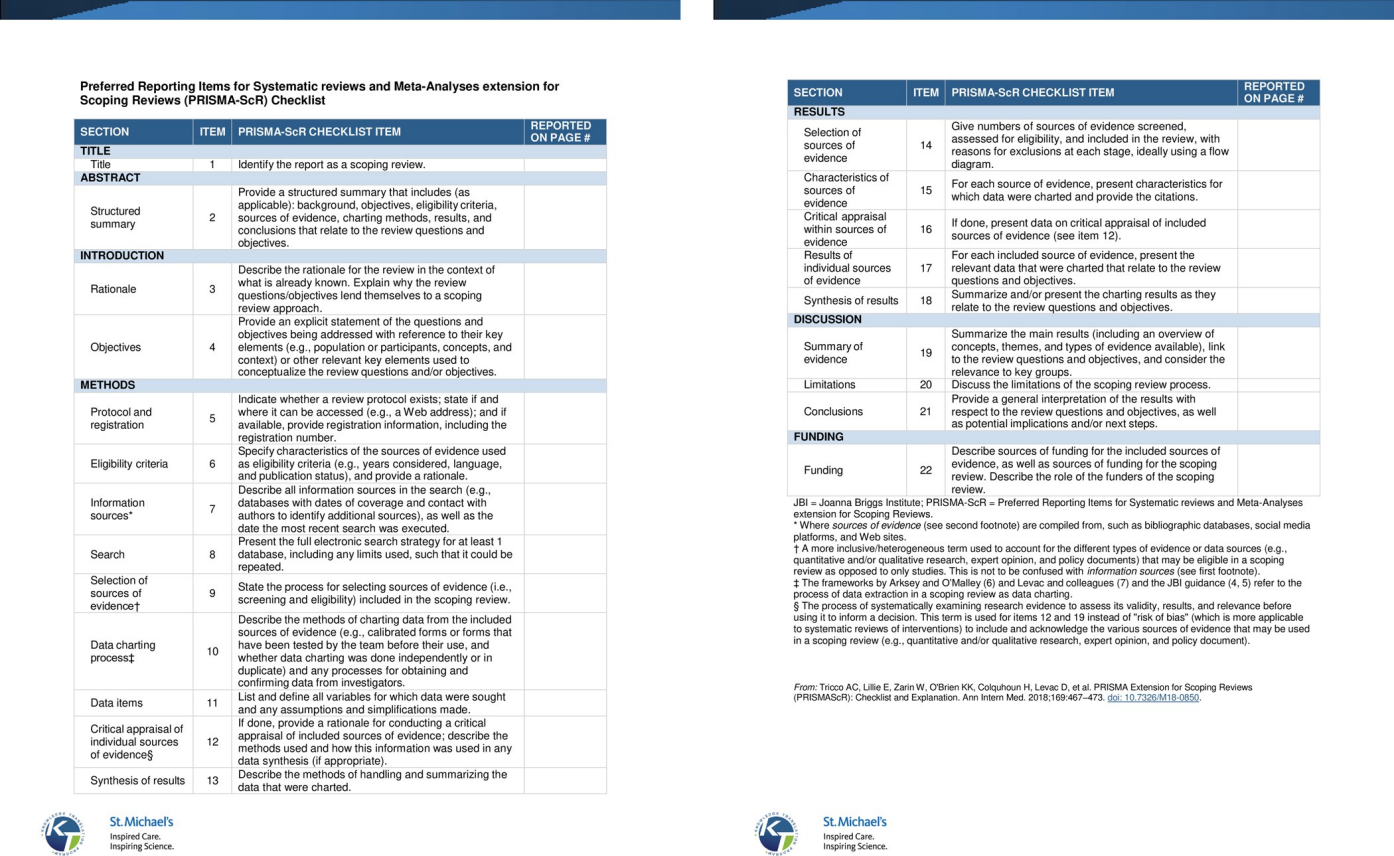
PRISMA-ScR [29].

The broad nature of our research question and the large volume of literature published around our topic identified a scoping review as a fitting methodology for conducting this review [25, 26]. The documentation process of systematic searching, screening and synthesis of literature will offer results that are trustworthy by using a method that is replicable [25, 26]. The study’s design will be guided by Arksey and O’Malley’s (2005) [25] methodological framework for conducting scoping reviews as refined by Levac *et al*. (2010) [27]. The Joanna Briggs Institute’s (JBI, updated 2020) [28] manual for conducting scoping reviews will guide this review, whilst data charting will follow the Preferred Reporting Items for Systematic reviews and Meta-Analyses extension for Scoping Reviews (PRISMA-ScR) reporting checklist (see Fig 1) [29]. Presented below is a non-linear framework which consists of five iterative stages to guide this study. It should be noted that the framework has an optional sixth stage which speaks to consulting with stakeholders however, this stage will be excluded for the purpose of this study as we will not consult with any stakeholders [25].

### Stage 1: Identifying the research question

As part of this study’s conceptual process, an informal literature search was conducted by the primary investigator (PI) to advise the development of the preliminary research question. The yield of the initial literature search was reviewed to generate a sense around the knowledge-gaps in the literature. Following the literature review, in accordance with the recommendations made by the JBI [28], we applied the “Population, Concept, and Context” (PCC) framework as a guide to frame our primary research question (see Table 1). It was important at this stage for the research team to define the key concepts and population [30]. We agreed on the following concepts as central to our study: *SAVA syndemic*, *violence*, *substance use*, *depression*, *anxiety*, *and ART adherence behaviour*.

**Table 1:** Population- concept-context framework [2,8].

Components	Definition	Study
Population	Important characteristics (including age and other qualifying factors)	People living with HIV who have initiated ART (no age restriction, includes at-risk populations)
Concepts	A clear definition of the key concepts (including the scope and breadth of the search)	ART adherence behaviour, violence, substance use, anxiety, depression, SAVA syndemic
Context	Broad (including cultural, geographic, gender, racial or population factors)	Sub-Sahara Africa (impoverished settings)

Our population of interest is PLWH who reside in the SSA region. We define substance use as any use or abuse of harmful or hazardous psychoactive substances [31], this includes alcohol, street drugs and prescription or over-the-counter drugs. It was agreed that violence was a wide-ranging factor with many facets, with violence conceptualised as inclusive of inter- and/or intrapersonal abuse i.e. interpersonal violence, sexual and gender-based violence (SGBV), community-based violence, emotional abuse, self-harm or any other vicarious experiences of violence or harm.

Similarly, poor mental health is a broad concept that includes multiple conditions and criteria. It has been found that depression is the most widespread mood disorder found among PLWH [23], and is often experienced in conjunction with substance use [32–34] and/or violence [35]. Anxiety was identified as an additional key mood disorder due to its regular co-occurrence with depression [36] and strong association with HIV [37], substance use [38] and violence, specifically domestic and SGBV [39]. Similar to Kumar and colleagues (2009) [40], we defined ART adherence as the accuracy to which a client’s behaviour compares with their recommended healthcare regime as discussed and agreed upon with their health care provider. Sub-Saharan Africa was chosen as the context of our study due to its disproportionately high burden of HIV in relation to the rest of the world [41]. Our preliminary research question concluded as: *“What is the impact of concurrent occurring SAVA syndemics and mood disorders*, *manifested as anxiety and depression*, *on ART adherence behaviour among PLWH in the SSA region*?*”*

Although scoping reviews do not require their explicit outcomes to be stated [26], we intend to address our primary research question through answering a series of secondary research questions (see Table 2 for intended outcomes), namely: (i) *How prevalent is the SAVA syndemic among PLWH on ART in SSA*?; (ii) *How prevalent is the coexistence of the SAVA syndemic and depression and/or anxiety among PLWH on ART in SSA*?; (iii) *What is the impact of the SAVA syndemic on ART adherence in SSA*?; (iv) *What is the impact of depression and/or anxiety on ART*?

**Table 2:** Research questions and intended outcomes.

**Primary research question**	**Primary outcomes**
What is the impact of concurrent occurring SAVA syndemics and mood disorders, manifested as anxiety and depression, on ART adherence behaviour among PLWH in the SSA region?	A conceptual syndemics model to inform the conceptualisation around the co-occurrence of SAVA syndemics and poor mental health among PLWH who have initiated ART (-who reside in the SSA region).
**Secondary research questions**	**Secondary outcomes**
How prevalent is the SAVA syndemic among PLWHA on ART in SSA?	Prevalence levels/descriptives of mutually occurring SAVA syndemics among PLWH who have initiated ART (-who reside in the SSA region).
How prevalent is the coexistence of the SAVA syndemic and depression and/or anxiety among PLWH on ART in SSA?	Prevalence levels/descriptives of mutually occurring SAVA syndemics and depression and/or anxiety among PLWH who have initiated ART (-who reside in the SSA region).
What is the impact of the SAVA syndemic on ART adherence in SSA?	Tabular mapping detailing the impact which SAVA syndemics has on ART adherence among PLWH who have initiated ART (-who reside in the SSA region).
What is the impact of depression and/or anxiety on ART adherence?	Tabular mapping detailing the impact of depression and/or anxiety on ART adherence among PLWH who have initiated ART ART (-who reside in the SSA region).

### Stage 2: Identify relevant studies

The PI developed the search strategy which was refined in collaboration with the research team. Six contextually relevant electronic databases were identified for the study and will be accessed through the University of the Western Cape’s library databases: *Academic search complete* (EbscoHost); *APA PsycArticles* (EbscoHost); *CINAHL* (EbscoHost); *MEDLINE* (EbscoHost); *SocINDEX* (EbscoHost); and *Web of Science*. Our Boolean search string was informed by our preliminary literature review–see Table 3 for the selected databases, search field and Boolean search string.

**Table 3:** Database, field and boolean search strings.

Databases	Field	Boolean combinations
*Academic search complete*	Title	HIV AND (violence OR abuse OR gender-based violence OR GBV OR intimate partner violence OR IPV AND depress* OR anxiety OR mood disorder*) OR (substance* OR drug* OR addict* OR people who inject drugs OR PWID* OR SAVA AND depress* OR anxiety OR mood disorder*) AND (association OR relationship) AND (antiretroviral OR antiretroviral therapy OR HAART AND adhere*) AND (sub Saharan Africa OR SSA OR Angola OR Benin OR Botswana OR Burkina Faso OR Burundi OR Cabo Verde OR Cameroon OR Central African Republic OR Chad OR Comoros OR Congo OR Cote d’Ivoire OR Equatorial Guinea OR Eritrea OR Ethiopia OR Gabon OR Gambia OR Ghana OR Guinea OR Guinea-Bissau OR Kenya OR Lesotho OR Liberia OR Madagascar OR Malawi OR Mali OR Mauritania OR Mauritius OR Mozambique OR Namibia OR Niger OR Nigeria OR Rwanda OR Sao Tome and Principe OR Senegal OR Seychelles OR Sierra Leone OR Somalia OR South Africa OR South Sudan OR Sudan OR Tanzania OR Togo OR Uganda OR Zambia OR Zimbabwe)
*APA PsycArticles*	Abstract	
*CINAHL*	Title	
*MEDLINE*	Title	
*SocINDEX*	Title	
*Web of science*	Title	

However, we acknowledge that our search string may change as we continue to refine our literature search as recommended by Levac *et al*. (2006) [30]. This study will include qualitative, quantitative and/or mixed-methods peer-reviewed publications, with literature published only in Afrikaans, English, Dutch, and French due to the language limitation of the research team. Furthermore, age will not be considered a limiter as HIV, violence, substance use and mood disorders, nor the impact thereof, are limited by age. Only literature published from the year 2010 till 2022 will be included in the search, we opted for a time limit that fell within the last decade to include findings that coincide with the widespread roll-out of ART, the test-and-treat initiative, and pre- and postexposure prophylaxis (PEP). This timeframe was identified to limit the potential for research that may be dated due to the aforementioned medical advancements. In addition, reference lists of articles included for full-text review will be hand searched to maintain the breadth of this review’s inclusivity [30]. All literature searches will be conducted by the research team.

### Stage 3: Study selection

**Fig 2:**
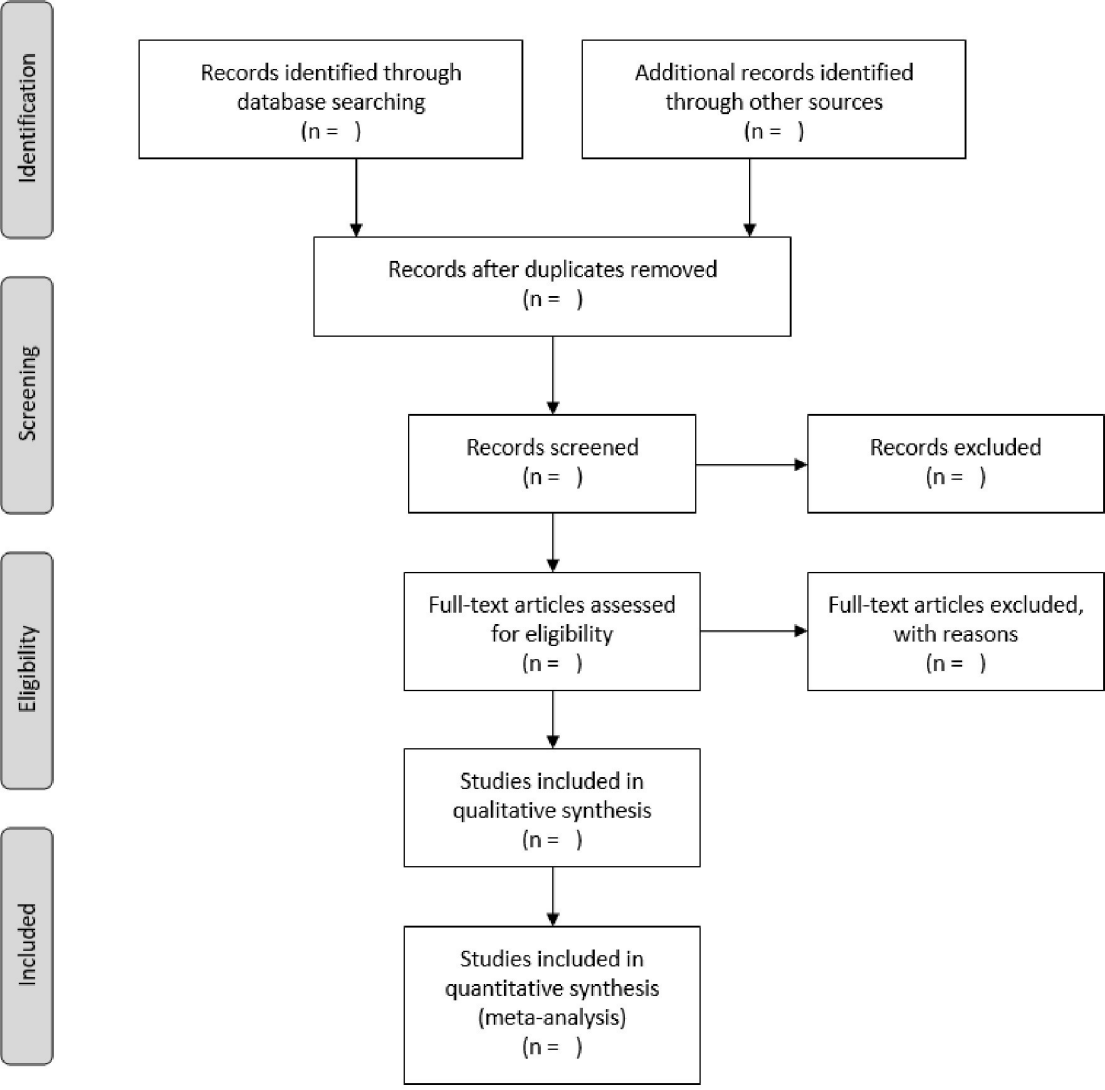
PRIMSA flowchart.

Adhering to the recommendations made by Levac *et al*. (2006) [30, 42], identifying articles will follow a transparent, iterative review process before finalising the inclusion and exclusion criteria. The team will meet regularly throughout the screening process to discuss the results and update the criteria should it be required. A refined literature search will be led by the PI and the yield of the search exported to Mendeley for removal of duplicate references. The reviewing process will consist of two phases: (1) title/abstract screening and (2) full text screening. In the first stage, the identified references will be sorted alphabetically by author and divided equally among the three reviewers, i.e. each reviewer will screen a third of the titles. Reference titles will be screened using the PCC mnemonic as a guideline, it should be noted that the inclusion criteria may be refined post-hoc [30]. Furthermore, in the event of a reference with an ambiguous title, the abstracts will be screened using the PCC framework [30, 42]. References that were excluded during phase one of Stage 3, will undergo a second round of review by an independent research assistant. The second phase of this stage involves the screening of the ‘full-text’ of the references that have been identified for inclusion during phase one. This includes reading the content of the identified references, and compare it to the scope of this study using a rubric conceptualised on the PCC approach and the PRISMA-ScR [30] reporting criteria [30, 42]. The literature elimination process will be captured and detailed using the PRISMA flowchart (Fig 2).

### Stage 4: Charting the data

Articles that are included will have their data extracted using an adapted version of the JBIs evidence details, characteristics and results extraction tool to address the objectives of this study; and follow Tricco *et al’s* (2018) 27-item PRISMA-ScR [30] reporting guideline to guide and chart the contextual factors that are relevant for this review. All customisations of the extraction instrument will strictly follow the guidelines set out by the JBI [28]. It is noteworthy to highlight that the extraction tool will be further refined following an iterative process meaning, at specific intervals the team will regroup and review the information captured by the instrument and reflect the relevance of the contents in relation to the study’s aims and objectives [30].

Extraction of the data will be done by the research team who will pilot a data charting exercise using the adapted instrument to familiarise themselves with, and review the rigor of the instrument. For this exercise, two publications will be randomly selected from the list of identified articles, with each researcher independently charting these articles [30]. This exercise will ensure that there is a mutual understanding of the data charting process and that the process remain aligned with the purpose of this study [30]. In the event that the exercise does not offer conclusive consensus around extracting the data, the tool will be amended as per recommendation and the exercise repeated.

### Stage 5: Collating, summarising and reporting the results

The study’s final stage will follow the PRISMA-ScR as a guideline to report our findings, this will occur across a three-phased step-wise process as suggested by Levac *et al*. (2006) [30]. Although it is acceptable for scoping reviews to report only on simple frequency counts i.e. concepts, populations, and characteristics [28], this study will further provide results as a summary of coded categories i.e. barriers to ART adherence which will be determined using descriptive qualitative content analysis [28]. Qualitative and quantitative data, i.e. frequency counts, will be descriptively mapped and coded into thematic domains that aligns with substance use, violence, and poor mental health in relation to ART adherence and where noted, document the interaction between these factors. During the second step we will follow the PRISMA-ScR guideline to report our results and outcomes as identified in step one, and map these details accordingly [30]. In step three we intend to provide an overview of our results and present this in a tabular descriptive format, as well as reflect on potential research gaps as identified through our literature review [28]. Lastly, the findings will be contextualised within the syndemics model of health, and summarised as a biosocial conceptual model to objectify the impact of co-occurring SAVA syndemics and poor mental health on ART adherence among PLWH residing in the SSA region (see Table 2: intended outcomes).

### Ethics and dissemination

This study will make use of secondary data that are available to the public and does not require additional ethics approval. We intend to publish our results in a peer-reviewed journal and disseminate our findings at appropriate conferences and seminars. We acknowledge that the limitations of this proposed scoping review are found within the parameters of our search criteria and database limiters including time span (2010–2022), language (Afrikaans, English, Dutch, and French only), exclusion of white or green papers, grey literature and any literature that may not have been identified within our selected databases.

## Discussion

This scoping review marks the first phase towards developing a model that will represent the synergistic and mutually reinforcing impact substance use, violence and mood disorders have on ART adherence behaviour. We foresee that the results will illuminate a broader foundation from which to build our understanding regarding the clustering and impact of the synergistic co-occurrence of the SAVA syndemic and mood disorders; and how these adverse health clusters perpetuate poor ART adherence behaviour, specifically among SSA populations.
